# Idiopathic, Pernicious, or Addison's Anæmia

**Published:** 1909-10-30

**Authors:** C. O. Hawthorne

**Affiliations:** Examiner in Medicine and Clinical Medicine in the University of Glasgow; Physician to the North-West London Hospital and the Central London Ophthalmic Hospital


### Hospital Clinics.

### IDIOPATHIC, PERNICIOUS, OR ADDISON'S ANÆMIA.

### II. NOTES ON TREATMENT.

By C. O. HAWTHORNE, M.D., M.R.C.P., Examiner in Medicine and Clinical Medicine in the University of Glasgow; Physician to the North-West London Hospital and the Central London Ophthalmic Hospital.

The treatment of pernicious anæmia, or progressive pernicious anæmia, as it is sometimes called, is by no means the hopeless task which the terms "progressive" and "pernicious" suggest. Indeed, one of the arguments which Dr. William Hunter advances in support of his proposal to abolish these epithets, is that they are inaccurate. Not only do they convey an ominous and depressing conviction to the patient's mind—on which score alone they are objectionable—but in doing this they have not the full warrant of facts. That severe and active cases of the disease occur, and that these often, and in spite of treatment, pass more or less rapidly to a fatal conclusion, cannot be denied. But this is not the whole of the picture; nor, indeed, is it the main part. The more important and more comprehensive statement is that, as seen in the majority of cases, pernicious anæmia is a disease which shows a very decided tendency towards improvement, though such improvement, unfortunately, is for the most part of but moderate duration and is followed by relapse. Still, the fact that the disease shows this natural effort towards recovery is an encouragement to the physician, and, without question, life may often be prolonged by judicious and persevering treatment. To attain success, however, it is essential that the patient shall accept direction not merely when he is manifestly prostrate and ill, but also in the quieter intervals between the more active phases of the disease. And, in addition, the therapeutic scheme must comprise something more than the routine administration of some favourite drug or drugs; rather it must include a complete survey and detailed ordering of the patient's habits and surroundings. Granted these conditions, the treatment of pernicious anæmia will be found in the majority of cases anything but a hopeless and unthankful task.

#### Essentials of Treatment.

Fortunately, although differences of opinion exist on minor points, most authorities are agreed on the essential methods of treatment necessary in this disease. These principles include the establishment of proper hygienic surroundings, the selection of a suitable diet, the destruction of septic conditions existing in the alimentary canal, and the administration of certain remedies of which the arsenic is the chief.

Hygiene, including an abundant supply of pure air and of sunshine, is of the first importance, and in many cases the kind of life now usually advised in this direction in phthisis pulmonalis may be recommended. Rest, also, is of high value, and, indeed, during the active and febrile stages of the disease the patient must be kept in bed. At all times over-exertion is prejudicial, and the same must be said of anxiety, mental strain, and worry. The feeding of the patient, at least in the more acute phases of the disease, is largely governed by the state of the stomach. Retching and vomiting at this stage are often troublesome features, and may restrict the food proposals to very simple limits; perhaps, for a time nothing but peptonised milk may be possible. Usually under full doses of bismuth, with or without a small addition of morphine, and with sinapisms over the epigastrium, the sickness can be kept in check, and then a more liberal dietary may be practised. But it is generally allowed that, with the disease showing decided activity, highly nitrogenous foods are unsuitable, and Dr. Lovell Gulland in particular is emphatic that so long as the blood count is low the diet should be restricted entirely to milk and farinaceous foods. As the stomach permits and the blood improves greater liberty may be allowed, and so far as seems reasonable the promptings of the patient's appetite may be followed. But plain rather than rich foods are to be advised, and the diet should contain a fair proportion of fats.

#### Cholesterin as an Aid in the Treatment.

In connection with the last statement, it may be noted that some physicians have claimed good results in pernicious anæmia from the administration of cholesterin in olive oil (3 grams in 100 daily); as a substitute for this, and better borne by the stomach, ¼ to ½ lb. butter and ½ to 1 litre of cream have been advised, the retention and assimilation of these large quantities of fat being promoted by the addition to them of a little brandy, and of a powder containing calcium carbonate and phosphate. This recommendation of fats has relation to a suggested explanation of the essential pathology of pernicious anæmia. The suggestion is that the red blood corpuscles formed in the bone-marrow have, in this disease, a stroma defective in materials allied to fats (lecithin, cholesterin), and that in consequence of this defect they readily undergo destruction; hence the remedial value of cholesterin, fats, and perhaps also of red bone-marrow. In similar fashion may be explained the therapeutic virtue of arsenic, for it may be suggested that by stimulating the bone-marrow arsenic leads to the production of red corpuscles having a normally constituted and therefore a stable and resistant stroma.

#### The Need for Antiseptics.

In the selection of medicines in the treatment of pernicious anæmia, everyone recognises the need for removing or neutralising septic states of the alimentary canal, the value of arsenic, and the uselessness or even harmfulness (except in certain special circumstances) of iron. On the value of bone-marrow, anti-streptococcic serum, and some other proposed remedies opinions differ widely.

Septic states of the mouth, stomach, and intestine lead to the production and absorption of poisons which exert a prejudicial influence on the bone-marrow, and therefore interfere with the source from which a favourable reaction in pernicious anæmia is to be anticipated. Hence the necessity for the careful and thorough use of antiseptic agents.

### Pay Attention to the Teeth.

Particular attention should be paid to the condition of the mouth and teeth; disinfectant mouth-washes should be employed, and should be rubbed with a soft brush into the gums and mucous membrane; the teeth should be thoroughly cleaned, and if there is dental caries the services of the dentist may be required. It must be remembered that asepsis of but limited distribution may, if continuous, be a cause of serious mischief by offering an unbroken opportunity for the entrance of hurtful material into the blood. To check septic processes in the gastric mucous membrane salicylic acid (10 grains in a cachet two or three times daily) or bismuth salicylate may be ordered; or lavage of the stomach may be practised, and this measure may exceptionally be needed when vomiting and retching are very troublesome. A mixture containing solution of pepsine with dilute hydrochloric acid is often of value as helping to replace the abnormal deficiency of the gastric juice. In many instances septic processes are active also in the intestine, as shown by abdominal discomfort, flatulence, and fœtor of the stools. These conditions may be met by calomel, corrosive sublimate, or salol, and a large enema of ordinary saline solution may be given every day. Another agent acting in the same direction is lacto-bacilline or some other form of "soured" milk.

### Drugs—Arsenic.

Arsenic as a remedy in pernicious anæmia was introduced by Dr. Byrom Bramwell and is now universally prescribed. Some physicians commence with small doses, rapidly push the remedy until the limits of tolerance are reached, and then reduce the amount by one-half; others teach that moderate doses are as effective as large ones. Again, while some authorities say that arsenic in small doses should be used throughout the whole course of the disease with only an occasional intermission, others advise the increasing use of the remedy until, as shown by the condition of the blood, a maximum effect has been produced, when they withdraw it and repeat it only on the first sign of a relapse. The argument in favour of the latter course is that continuous stimulation may exhaust the blood-regenerating power of the bone-marrow. Whatever plan is adopted, it is certain that different individuals require different doses, and that here, as elsewhere, effective prescribing requires a study of the idiosyncrasies of the patient rather than a pedantic adherence to the announcements of a pharmacopœia. It is also of importance that every patient should be kept under frequent observation in order that the blood may be examined at regular intervals and the first beginnings of a relapse be detected and treated. As for the form in which arsenic should be prescribed, this is probably not of much consequence. Fowler's solution (some omit the tincture of lavender) is perhaps most generally ordered, but the liquor arsenici hydrochloricus or sometimes Donovan's solution may be employed; for hypodermic injection various formulæ are prepared (see Martindale's "Extra Pharmacopœia").

### Red Bone Marrow.

In addition to arsenic, red bone-marrow is a remedy for which something like specific value has been claimed. It was first used by Sir Thos. Fraser, and there are many testimonies to its value; tablets and a glycerine extract are available, or the marrow (2 to 4 ounces daily) may be given fresh in the form of sandwiches. Dr. Gulland has no faith in the merits of bone-marrow, nor does he believe in the virtues of anti-streptococcic serum in the treatment of pernicious anæmia. On the other hand, the serum is strongly urged by Dr. Wm. Hunter. It may be given in doses of 5 to 10 c.c. and repeated at intervals of two to four days; it appears to be of value in the more severe phases of the disease. Iron, as already stated, is not generally indicated in pernicious anæmia, and indeed is often harmful. But sometimes, when, under other treatment, the condition of the blood has decidedly improved and examination shows a low colour index, there is opportunity for the employment of iron. Cannabis indica, calcium chloride, and many other drugs have been announced as successful in individual cases, but have failed to establish the position claimed for them; and transfusion, which a few years ago was strongly advised by several physicians, appears to have fallen into complete disuse.

### Conclusions.

Speaking generally, it may be said that pernicious anæmia includes some cases which resist all remedies, and which, in spite of treatment, terminate more or less rapidly in death; that in the majority of cases, on the other hand, there are remissions with a very decided movement towards recovery; and that, unfortunately, remission is almost invariably followed by relapse. These facts in the natural history of the disease help to explain the conflict of opinion and statement in reference to the value of various remedies. In the most acute cases all remedies fail to justify their reputation, while any drug administered when a natural remission is impending is apt to receive a degree of credit which further experience fails to sustain. It is, however, certain that in most cases, provided the diagnosis is made early, much may be accomplished by attention to hygiene, by getting rid of septic states of the alimentary canal, and by the administration of arsenic either alone or in association with hypophosphites and other tonics. In addition, bone-marrow and anti-streptococcic serum are certainly worthy of trial, and iron, although not generally suitable, cannot be altogether excluded from the scheme of treatment.

